# Nomogram-based prediction of in-hospital mortality and embolic events in infective endocarditis

**DOI:** 10.3389/fmicb.2026.1923055

**Published:** 2026-09-07

**Authors:** Chang Liu, Jun Fan, Bingbo Yu, Weijie Li, Shaohua Wang, Bin Zhao, Shaoling Luo, Senwei Jiang, Xiaoxuan Xu

**Affiliations:** 1 Department of Cardiology, Guangzhou First People’s Hospital, School of Medicine, South China University of Technology, Guangzhou, China; 2 Department of Cardiology, Guangzhou First People’s Hospital, Guangzhou Medical University, Guangzhou, China; 3 Department of Gynecology, The Third Affiliated Hospital of Sun Yat-sen University, Guangzhou, China; 4 Department of Hematology, Guangzhou First People’s Hospital, School of Medicine, South China University of Technology, Guangzhou, China; 5 Department of Hematology, Guangzhou First People’s Hospital, Guangzhou Medical University, Guangzhou, China

**Keywords:** embolic events, infective endocarditis, nomogram, predictive modeling, risk stratification, systemic inflammatory indices

## Abstract

**Background:**

Infective endocarditis (IE) remains a life-threatening infection associated with high short-term mortality and frequent thromboembolic complications. Reliable bedside tools for early and individualized risk stratification remain limited. This study aimed to identify predictors of in-hospital mortality and new embolic events in adult patients with IE and to develop and internally validate nomogram-based prediction models.

**Methods:**

This retrospective cohort study included consecutive adult patients with definite IE admitted to Guangzhou First People’s Hospital between January 2020 and December 2025, according to the 2023 Duke–ISCVID criteria. Six routinely available variables–age, vegetation size, white blood cell (WBC) count, log_2_-transformed N-terminal pro-B-type natriuretic peptide (NT-proBNP), log_2_-transformed systemic immune-inflammation index (SII), and Prognostic Nutritional Index (PNI)–were entered into multivariable logistic regression models. Nomograms were constructed and evaluated using receiver operating characteristic (ROC) curve analysis, calibration plots with 1,000 bootstrap resamples, and decision curve analysis (DCA).

**Results:**

A total of 62 patients were included. In-hospital mortality occurred in 8 patients (12.9%) and new embolic events in 13 patients (21.0%). For the embolic model, echocardiographic vegetation size and WBC count were independently associated with embolic risk, yielding an AUC of 0.882. For the mortality model, log_2_-NT-proBNP was independently associated with in-hospital death, with the nomogram demonstrating a discriminative AUC of 0.806. Calibration analysis confirmed consistency between the predicted and observed probabilities. DCA indicated a positive net clinical benefit across threshold probabilities of 1%–42% for mortality and 5%–66% for embolic events.

**Conclusion:**

NT-proBNP was independently associated with in-hospital mortality, whereas vegetation size and WBC count were key predictors of embolic risk in patients with IE. These nomogram-based models provide practical tools for individualized bedside risk assessment. External validation in larger multicenter cohorts is warranted.

## 1 Introduction

Infective endocarditis (IE) remains a devastating cardiovascular infection characterized by rapid endocardial destruction, local tissue invasion, and life-threatening systemic complications (Cahill and Prendergast, 2016). Despite advances in antimicrobial therapy, diagnostic imaging, and cardiac surgery, the clinical burden of IE has not fundamentally declined over the past decades (Baddour et al., 2015). Globally, infective endocarditis remains a highly fatal disease, with an overall mortality rate of approximately 25% (Nishimura et al., 2014). Additionally, embolic complications–most frequently affecting the central nervous system–are reported in 22%–50% of patients (Bayer et al., 1998; Vilacosta et al., 2002). Such embolic events contribute substantially to morbidity and mortality.

The epidemiology of IE has evolved markedly, characterized by a steady decline in rheumatic heart disease alongside a prominent rise in degenerative valvular diseases, prosthetic valve implantations, cardiovascular implantable electronic device (CIED) infections, healthcare-associated exposures, and intravenous drug use (Delgado et al., 2023). The clinical presentation of IE has become increasingly atypical, with a growing proportion of patients exhibiting clusters of multiple comorbidities, blunted febrile responses, and non-specific or even absent symptoms, all of which frequently contribute to prolonged diagnostic delays (Habib et al., 2019). Adverse outcomes of IE–most notably short-term mortality and major embolic events–remain heavily associated with prolonged hospitalization, irreversible organ damage, and substantial healthcare resource utilization (Cahill et al., 2017). Given such highly heterogeneous and often insidious clinical trajectories, early and accurate risk stratification is paramount to identify highly vulnerable individuals who may benefit from intensified hemodynamic monitoring or expedited surgical evaluation (Delgado et al., 2023; Santos-Patarroyo et al., 2025).

Predicting short-term mortality in acute infective endocarditis (IE) remains an intricate and critical clinical challenge (Cahill and Prendergast, 2016). During the acute phase, the host response is characterized by “immunothrombosis” –a pathophysiological hallmark where innate immunity and the microvascular coagulation cascade converge to stabilize or expand the infected valvular lesion (Engelmann and Massberg, 2013). However, accumulating clinical evidence suggests that although immunothrombotic and systemic inflammatory markers are universally elevated, fatal outcomes in these patients are frequently dominated by myocardial wall stress, acute valvular regurgitation, and subsequent hemodynamic exhaustion, rather than by the localized inflammatory insult *per se* (Thuny et al., 2012). In this specific context, N-terminal pro-B-type natriuretic peptide (NT-proBNP)–a biomarker synthesized and released by cardiomyocytes in response to progressive ventricular stretch–has emerged as a highly sensitive surrogate for secondary heart failure and impending cardiogenic shock (Bertolino et al., 2022).

However, expanding evidence suggests that the practical prognostic performance of these hemodynamic markers might be significantly modulated by the host’s baseline systemic resilience (Evans et al., 2021). The Prognostic Nutritional Index (PNI), comprehensively calculated from serum albumin levels and total lymphocyte count, reflects both steady-state nutritional status and sustained immunological capacity (Okada et al., 2017). While the neutrophil-to-lymphocyte ratio (NLR) primarily tracks acute-phase stress, the systemic immune-inflammation index (SII)–which integrates platelet, neutrophil, and lymphocyte count–offers a broader window into immunothrombotic complexity, capturing the interplay between platelet-driven thrombosis and neutrophil-mediated inflammation (Liu et al., 2022; Qin et al., 2025). In contrast, the PNI reflects the host’s long-term metabolic and immune reserve. Mechanistically, severe hypoalbuminemia and progressive lymphopenia are frequently observed during intractable infections, serving as key indicators of metabolic depletion and stress-induced immune exhaustion (Hotchkiss et al., 2013). Although cardiac stress biomarkers and nutritional frameworks have each been independently associated with outcomes in critical illness, their integrated utility for multidimensional risk stratification in adult IE remains largely unexplored (Bertolino et al., 2022; Delgado et al., 2023). Together, PNI (systemic reserve) and SII (immunothrombotic activity) may capture complementary dimensions of host pathophysiology.

Concurrently, prediction of new embolic complications requires a distinct, multidimensional pathophysiological approach (Thuny et al., 2005; Santos-Patarroyo et al., 2025). Embolic events, particularly ischemic cerebral infarctions, remain major causes of long-term disability and early mortality in adult patients with definitive IE (García-Cabrera et al., 2013). Historically, embolic risk assessment has relied almost exclusively on macroscopic echocardiographic vegetation size (Mügge et al., 1989). However, clinical paradoxes frequently arise where some patients with large vegetations never embolize, whereas others with small lesions suffer catastrophic strokes, strongly suggesting that embolization is not merely a passive mechanical phenomenon (Thuny et al., 2005). During the acute infective phase, a prominent elevation in peripheral white blood cell (WBC) count is observed, a systemic response predominantly driven by the urgent mobilization of circulating neutrophils (Manz and Boettcher, 2014). These overactivated neutrophils aggressively infiltrate the local vegetation matrix, actively releasing proteolytic enzymes and organizing neutrophil extracellular traps (NETs) that destabilize the structural fibrin scaffold and promote embolic fragmentation (Brinkmann et al., 2004; Meyers et al., 2023). Whether the net effect of vegetation size and peripheral WBC count on embolic risk remains independent after adjusting for age, cardiac stress (NT-proBNP), immunothrombotic complexity (SII), and host nutritional reserve (PNI) has not been established, and a unified predictive panel integrating these multidimensional domains is still lacking (Delgado et al., 2023; Bertolino et al., 2025).

To explore potential solutions to these clinical gaps, we developed two hypothesis-generating, routine-parameter-based nomograms tailored for hospitalized adult patients with definite IE. Both predictive frameworks incorporated a comprehensive panel of six baseline covariates: age, vegetation size, WBC count, log_2_-NT-proBNP, log_2_-SII, and PNI. The first model predicts in-hospital all-cause mortality, for which log_2_-NT-proBNP emerged as the sole statistically significant independent predictor; the second predicts new embolic risk, which is independently driven by vegetation size and WBC count. By translating these multivariable regression formulas into intuitive visual nomograms and evaluating their clinical utility with decision curve analysis (DCA), we aim to provide a preliminary exploratory framework for individualized bedside risk stratification.

## 2 Materials and methods

### 2.1 Study population and patient selection

This retrospective cohort study screened consecutive adult patients diagnosed with IE who were admitted to Guangzhou First People’s Hospital between January 2020 and December 2025. The diagnosis of IE was established according to the updated 2023 Duke–ISCVID criteria (Fowler et al., 2023).

Inclusion criteria were: (1) age ≥18 years and (2) a definite diagnosis of IE per the 2023 Duke–ISCVID criteria. Patients were excluded if key clinical, echocardiographic, or laboratory parameters required for the baseline panels and multivariable models were missing. Specifically, the major reasons for exclusion were the absence of complete records for NT-proBNP, procalcitonin (PCT), serum albumin, hemoglobin, platelet count, neutrophil count, or lymphocyte count.

**FIGURE 1:**
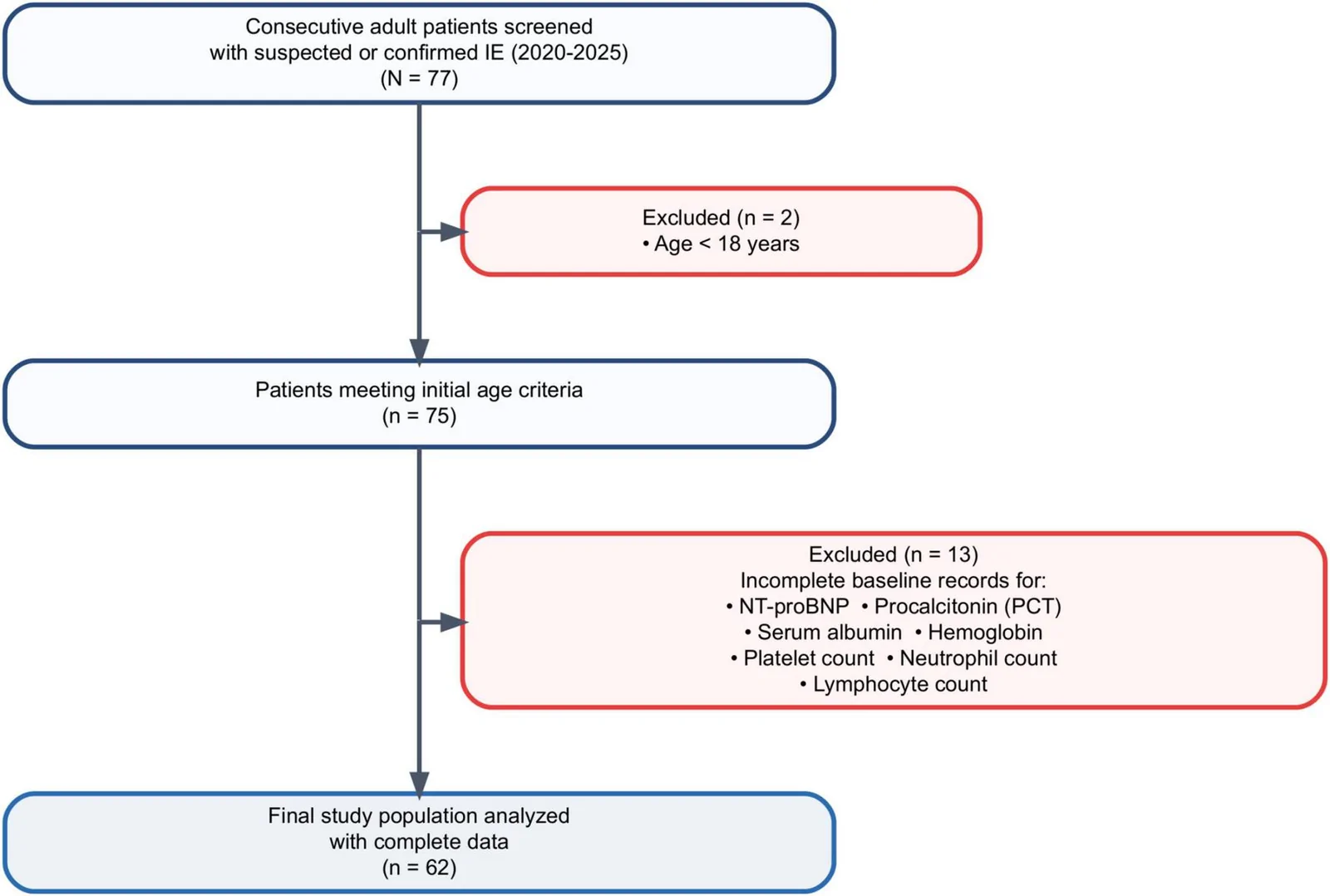
Flow diagram of patient selection. A total of 75 patients with infective endocarditis were initially screened. Thirteen were excluded due to incomplete baseline data, leaving 62 patients in the final study cohort.

A total of 75 patients were initially screened. Thirteen patients were excluded due to missing key baseline laboratory data, including 6 patients with missing NT-proBNP values, 6 patients with missing PCT values, and 1 patient with both NT-proBNP and PCT missing. Thus, 62 patients with complete key clinical and laboratory data were included in the final analysis (Figure 1). The study was conducted in accordance with the Declaration of Helsinki and approved by the Ethics Committee of Guangzhou First People’s Hospital (Approval No. K-2026-110-01). The requirement for written informed consent was waived due to the retrospective design.

### 2.2 Data collection and variable definitions

Baseline demographic, clinical, laboratory, and echocardiographic data were extracted from the electronic medical record system by two independent investigators. Demographic and clinical variables included age, sex, hypertension, diabetes mellitus, chronic kidney disease, liver cirrhosis, prior IE, prosthetic valve status, intravenous drug use, pre-admission antibiotics use, NYHA functional class, septic shock, and surgical intervention during hospitalization.

All laboratory measurements were obtained from the first blood sample collected within 24 h of admission, before major therapeutic interventions. Laboratory variables included WBC count, absolute neutrophil count, lymphocyte count, hemoglobin, platelet count, C-reactive protein (CRP), PCT, serum albumin, creatinine, myoglobin, creatine kinase MB (CK-MB), and high-sensitivity cardiac troponin I (hs-cTnI). Advanced inflammatory indices, including the neutrophil-to-lymphocyte ratio (NLR), platelet-to-lymphocyte ratio (PLR), and systemic immune-inflammation index (SII), were also calculated.

Echocardiographic data were obtained from transthoracic or transesophageal echocardiography performed during the initial phase of hospitalization, and included maximal vegetation size (longest linear dimension), severe valvular regurgitation, and myocardial abscess formation.

The Prognostic Nutritional Index (PNI) was calculated as (Onodera et al., 1984):


P⁢N⁢I=10×serum⁢albumin⁢(g/dL)+0.005×total⁢lymphocyte

$$\text{count}\left(/{\mathrm{mm}}^{3}\right)$$

The SII was calculated as (Hu et al., 2014):


$$\text{SII}=\frac{\text{platelet}\,\text{count}\times \text{neutrophil}\,\text{count}}{\text{lymphocyte}\,\text{count}}$$

Because NT-proBNP, hs-cTnI, and SII values exhibited heavily right-skewed distributions, they were log_2_-transformed before regression analyses to achieve statistical stability.

### 2.3 Study outcomes

The primary outcome was all-cause in-hospital mortality. The secondary outcome was new embolic events during hospitalization, defined as clinically or radiologically confirmed cerebral embolism, peripheral arterial embolism, splenic or renal infarction, or other systemic embolic complications verified by computed tomography, magnetic resonance imaging, or vascular ultrasound. Patients with pre-existing embolic lesions were not counted. Factors associated with surgical intervention were also explored descriptively.

### 2.4 Statistical analysis and model development

Analyses were performed using R software (version 4.5.3). The reporting followed the TRIPOD statement (Collins et al., 2015) (Supplementary Material 1). Continuous variables were assessed for normality using the Shapiro–Wilk test. Baseline continuous variables are presented as mean ± SD and were compared using Student’s *t*-test or the Mann–Whitney U test depending on data distribution. Categorical variables are presented as frequencies and percentages, and compared using the χ^2^ or Fisher’s exact test.

Univariate logistic regression was initially conducted across clinical parameters. Based on the study objectives and the clinical and biological relevance of the candidate variables, six prespecified predictors–age, vegetation size, WBC count, log2-NT-proBNP, log2-SII, and PNI–were entered simultaneously into the multivariable logistic regression models for both in-hospital mortality and new embolic events. Concurrently, for the exploratory analysis of factors associated with undergoing surgical intervention, a multivariable model was constructed incorporating age, WBC count, hemoglobin, log_2_-NT-proBNP, log_2_-SII, and PNI, selected *a priori* based on their clinical and biological relevance to the study objectives. These variables were prespecified rather than selected solely according to univariable statistical significance, and the resulting models were considered exploratory and hypothesis-generating given the limited number of outcome events.

Nomograms were constructed based on the final multivariable logistic regression models. Discrimination was evaluated using receiver operating characteristic (ROC) curves and the area under the curve (AUC); optimal cut-off values were determined by the Youden index, with corresponding sensitivity and specificity. Calibration was assessed using calibration plots generated from 1,000 bootstrap resamples. Given the relatively small sample size and limited number of outcome events, model performance was interpreted cautiously, with emphasis on the exploratory nature of the predictive model. DCA was performed to evaluate net clinical benefit compared with “treat-all” and “treat-none” strategies across a range of threshold probabilities (Vickers and Elkin, 2006). All tests were two-sided; *p* < 0.05 was considered statistically significant.

## 3 Results

### 3.1 Baseline characteristics and factors associated with surgical intervention

A total of 62 patients with confirmed IE were included (Figure 1). The mean age was 58 ± 15 years, and 39 patients (62.9%) were male. During hospitalization, 25 patients (40.3%) underwent cardiac surgery, 8 (12.9%) died, and 13 (21.0%) experienced new embolic events (Table 1).

**TABLE 1:** Baseline characteristics of adult patients with infective endocarditis stratified by clinical interventions and outcomes.

Characteristic	Overall (*N* = 62)	No surgery (*n* = 37)	Surgery (*n* = 25)	*P*-value (surgery)	No embolism (*n* = 49)	Embolism (*n* = 13)	*P*-value (embolism)	Survived (*n* = 54)	Deceased (*n* = 8)	*P*-value (mortality)
Age (years) [mean ± SD]	58 ± 15	64 ± 14	50 ± 13	**<0.001**	58 ± 16	57 ± 13	0.86	58 ± 15	59 ± 20	0.92
Gender [*n* (%)]				0.79			0.52			0.13
Female	23 (37.1%)	13 (35.1%)	10 (40.0%)		17 (34.7%)	6 (46.2%)		18 (33.3%)	5 (62.5%)	
Male	39 (62.9%)	24 (64.9%)	15 (60.0%)		32 (65.3%)	7 (53.8%)		36 (66.7%)	3 (37.5%)	
Weight (kg) [mean ± SD]	60 ± 11	61 ± 11	60 ± 12	0.68	60 ± 11	62 ± 12	0.58	61 ± 11	56 ± 13	0.23
Height (cm) [mean ± SD]	167 ± 9	168 ± 8	165 ± 10	0.62	166 ± 9	168 ± 10	0.86	166 ± 9	174	0.38
Comorbidities and history [n (%)]										
Prior IE	1 (1.6%)	1 (2.7%)	0 (0.0%)	>0.99	0 (0.0%)	1 (7.7%)	0.21	1 (1.9%)	0 (0.0%)	>0.99
Prosthetic valve	4 (6.5%)	4 (10.8%)	0 (0.0%)	0.14	3 (6.1%)	1 (7.7%)	>0.99	4 (7.4%)	0 (0.0%)	>0.99
IVDU	0 (0.0%)	0 (0.0%)	0 (0.0%)	>0.99	0 (0.0%)	0 (0.0%)	>0.99	0 (0.0%)	0 (0.0%)	>0.99
Diabetes mellitus	4 (6.5%)	3 (8.1%)	1 (4.0%)	0.64	3 (6.1%)	1 (7.7%)	>0.99	4 (7.4%)	0 (0.0%)	>0.99
Hypertension	22 (35.5%)	19 (51.4%)	3 (12.0%)	**0.002**	18 (36.7%)	4 (30.8%)	0.76	20 (37.0%)	2 (25.0%)	0.70
Chronic kidney disease	8 (12.9%)	7 (18.9%)	1 (4.0%)	0.13	8 (16.3%)	0 (0.0%)	0.19	8 (14.8%)	0 (0.0%)	0.58
Liver cirrhosis	2 (3.2%)	1 (2.7%)	1 (4.0%)	>0.99	1 (2.0%)	1 (7.7%)	0.38	2 (3.7%)	0 (0.0%)	>0.99
Clinical presentation [n (%)]										
Pre-admission antibiotics	46 (74.2%)	29 (78.4%)	17 (68.0%)	0.39	33 (67.3%)	13 (100.0%)	**0.015**	39 (72.2%)	7 (87.5%)	0.67
Blood culture positivity	32 (51.6%)	20 (54.1%)	12 (48.0%)	0.8	26 (53.1%)	6 (46.2%)	0.76	29 (53.7%)	3 (37.5%)	0.47
Pathogen type [*n* (%)]				0.50			0.84			0.57
Culture-negative	30 (48.4%)	17 (45.9%)	13 (52.0%)		23 (46.9%)	7 (53.8%)		25 (46.3%)	5 (62.5%)	
*Staphylococcus aureus*	4 (6.5%)	3 (8.1%)	1 (4.0%)		3 (6.1%)	1 (7.7%)		3 (5.6%)	1 (12.5%)	
*Streptococcus* spp.	17 (27.4%)	8 (21.6%)	9 (36.0%)		13 (26.5%)	4 (30.8%)		16 (29.6%)	1 (12.5%)	
*Enterococcus* spp.	5 (8.1%)	4 (10.8%)	1 (4.0%)		4 (8.2%)	1 (7.7%)		5 (9.3%)	0 (0.0%)	
Other pathogens	6 (9.7%)	5 (13.5%)	1 (4.0%)		6 (12.2%)	0 (0.0%)		5 (9.3%)	1 (12.5%)	
Echocardiographic findings										
Vegetation size (mm) [mean ± SD]	13.7 ± 5.9	13.5 ± 5.9	14.1 ± 5.9	0.8	12.8 ± 5.4	18.6 ± 6.1	**0.011**	14.1 ± 6.0	11.7 ± 4.8	0.41
Perivalvular abscess [*n* (%)]	4 (6.5%)	1 (2.7%)	3 (12.0%)	0.29	3 (6.1%)	1 (7.7%)	>0.99	4 (7.4%)	0 (0.0%)	>0.99
Severe regurgitation [*n* (%)]	41 (66.1%)	21 (56.8%)	20 (80.0%)	0.10	33 (67.3%)	8 (61.5%)	0.75	35 (64.8%)	6 (75.0%)	0.71
Severity indices										
NYHA Functional class [*n* (%)]				0.4			0.86			**0.04**
Class I	8 (12.9%)	3 (8.1%)	5 (20.0%)		7 (14.3%)	1 (7.7%)		8 (14.8%)	0 (0%)	
Class II	27 (43.5%)	15 (40.5%)	12 (48.0%)		21 (42.9%)	6 (46.2%)		26 (48.1%)	1 (12.5%)	
Class III	17 (27.4%)	12 (32.4%)	5 (20.0%)		14 (28.6%)	3 (23.1%)		13 (24.1%)	4 (50.0%)	
Class IV	10 (16.1%)	7 (18.9%)	3 (12.0%)		7 (14.3%)	3 (23.1%)		7 (13.0%)	3 (37.5%)	
Septic shock [*n* (%)]	6 (9.7%)	3 (8.1%)	3 (12.0%)	0.68	3 (6.1%)	3 (23.1%)	0.10	4 (7.4%)	2 (25.0%)	0.17
Laboratory parameters										
WBC count (×10^9^/L) [mean ± SD]	11.4 ± 5.2	12.6 ± 5.4	9.8 ± 4.4	**0.035**	10.5 ± 4.3	15.0 ± 6.8	**0.026**	11.5 ± 5.3	11.1 ± 4.6	0.94
Neutrophil count (×10^9^/L) [mean ± SD]	9.4 ± 5.1	10.6 ± 5.3	7.5 ± 4.4	**0.013**	8.5 ± 4.3	12.7 ± 6.7	**0.043**	9.4 ± 5.2	8.9 ± 4.8	0.91
Lymphocyte count (×10^9^/L) [mean ± SD]	1.21 ± 0.64	1.07 ± 0.60	1.42 ± 0.65	**0.024**	1.23 ± 0.64	1.12 ± 0.67	0.53	1.18 ± 0.64	1.40 ± 0.66	0.44
Hemoglobin (g/L) [mean ± SD]	104 ± 24	98 ± 25	113 ± 18	**0.021**	105 ± 25	99 ± 20	0.40	103 ± 24	115 ± 22	0.25
Platelet count (×10^9^/L) [mean ± SD]	221 ± 105	219 ± 115	223 ± 90	0.93	225 ± 106	202 ± 103	0.70	218 ± 98	238 ± 150	0.92
CRP (mg/L) [mean ± SD]	82 ± 75	102 ± 79	50 ± 57	**0.006**	71 ± 67	123 ± 93	**0.045**	84 ± 74	70 ± 91	0.37
Procalcitonin (ng/mL) [mean ± SD]	7 ± 22	8 ± 23	5 ± 20	**0.012**	8 ± 24	3 ± 5	0.34	5 ± 19	15 ± 35	0.65
Albumin (g/L) [mean ± SD]	31.0 ± 5.3	29.0 ± 5.1	34.1 ± 4.0	**<0.001**	31.7 ± 5.3	28.6 ± 4.8	0.058	31.0 ± 5.4	31.6 ± 4.6	0.71
Creatinine (μmol/L) [mean ± SD]	144 ± 173	183 ± 214	88 ± 37	**0.018**	160 ± 192	87 ± 24	0.39	149 ± 181	112 ± 101	0.20
Myoglobin (ng/mL) [mean ± SD]	259 ± 795	239 ± 690	304 ± 1018	0.074	305 ± 890	88 ± 124	0.84	201 ± 628	581 ± 1430	0.84
CK-MB (ng/mL) [mean ± SD]	6 ± 13	5 ± 11	8 ± 16	0.62	5 ± 12	8 ± 15	0.62	6 ± 12	8 ± 17	0.29
hs-cTnI (ng/mL) [mean ± SD]	2.07 ± 11.13	0.67 ± 1.85	5.21 ± 19.96	0.16	2.31 ± 12.49	1.16 ± 2.72	0.16	0.58 ± 1.72	10.22 ± 28.20	0.23
Advanced biomarkers and indices										
log_2_-NT-proBNP [mean ± SD]	10.56 ± 2.43	11.02 ± 2.54	9.89 ± 2.13	0.081	10.43 ± 2.62	11.04 ± 1.52	0.43	10.37 ± 2.51	11.88 ± 1.21	0.068
NLR [Mean ± SD]	11 ± 10	14 ± 12	7 ± 5	**0.003**	10 ± 10	16 ± 11	**0.048**	12 ± 11	9 ± 6	0.73
PLR [mean ± SD]	244 ± 236	279 ± 276	192 ± 149	0.17	233 ± 182	283 ± 383	0.42	247 ± 237	223 ± 239	0.17
log_2_-SII [mean ± SD]	10.52 ± 1.36	10.90 ± 1.25	9.97 ± 1.36	**0.013**	10.39 ± 1.35	11.02 ± 1.33	0.24	10.57 ± 1.31	10.23 ± 1.77	0.41
PNI [mean ± SD]	37 ± 7	34 ± 7	41 ± 6	**<0.001**	38 ± 7	34 ± 7	0.10	37 ± 7	39 ± 6	0.59

Data are presented as mean ± SD for continuous variables or as number (percentage) for categorical variables. Significant *P*-values (<0.05) are indicated in bold. Statistical comparisons were performed using the Wilcoxon rank-sum test, Student’s *t*-test, Chi-square test, or Fisher’s exact test as appropriate based on data distribution. The SD for height in the deceased group was omitted due to a single available recording (*n* = 1). CK-MB, creatine kinase MB; CRP, C-reactive protein; Hb, hemoglobin; hs-cTnI, high-sensitivity cardiac troponin I; IE, infective endocarditis; IVDU, intravenous drug user; log_2_-NT-proBNP, log_2_-transformed N-terminal pro-B-type natriuretic peptide; log_2_-SII, log_2_-transformed systemic immune-inflammation index; NLR, neutrophil-to-lymphocyte ratio; NYHA, New York Heart Association; PLR, platelet-to-lymphocyte ratio; PLT, platelet count; PNI, prognostic nutritional index; WBC, white blood cell.

Baseline characteristics stratified by surgical intervention are shown in Table 1. Surgically treated patients were younger (50 ± 13 vs. 64 ± 14 years, *p* < 0.001) and had a lower prevalence of hypertension (12.0% vs. 51.4%, *p* = 0.002). They also had higher serum albumin (34.1 ± 4.0 vs. 29.0 ± 5.1 g/L, *p* < 0.001) and higher PNI (41 ± 6 vs. 34 ± 7, *p* < 0.001). WBC count (9.8 ± 4.4 vs. 12.6 ± 5.4 × 10^9^/L, *p* = 0.035) and CRP levels (50 ± 57 vs. 102 ± 79 mg/L, *p* = 0.006) were lower in the surgical group.

To explore factors associated with surgical intervention, a multivariable logistic regression model was constructed using the representative baseline panel (retaining age, WBC count, hemoglobin, log_2_-NT-proBNP, log_2_-SII, and PNI; vegetation size was omitted due to a lack of univariate association, *p* = 0.752). As shown in Table 2, the multivariable analysis revealed that younger age (OR = 0.940, 95% CI: 0.889–0.986, *p* = 0.019) and a higher PNI (OR = 1.163, 95% CI: 1.022–1.353, *p* = 0.032) were independently associated with undergoing cardiac surgery during hospitalization.

**TABLE 2:** Univariate and multivariable logistic regression analyses for factors associated with surgical intervention.

Variable	Univariate analysis		Multivariable analysis	
	OR (95% CI)	*P*-value	OR (95% CI)	*P*-value
Age (years)	0.929 (0.883, 0.968)	**0.001**	0.940 (0.889, 0.986)	**0.019**
Weight (kg)	0.989 (0.937, 1.043)	0.692	–	–
Vegetation size (mm)	1.015 (0.924, 1.116)	0.752	–	–
WBC count (×10^9^/L)	0.883 (0.772, 0.988)	**0.045**	0.983 (0.818, 1.171)	0.846
Neutrophil count (×10^9^/L)	0.867 (0.754, 0.973)	**0.027**	–	–
Lymphocyte count (×10^9^/L)	2.475 (1.089, 6.101)	**0.037**	–	–
Hemoglobin (g/L)	1.029 (1.005, 1.057)	**0.023**	1.016 (0.986, 1.050)	0.305
Platelet count (×10^9^/L)	1.000 (0.995, 1.005)	0.877	–	–
CRP (mg/L)	0.987 (0.975, 0.997)	**0.022**	–	–
Procalcitonin (ng/mL)	0.992 (0.957, 1.016)	0.559	–	–
Albumin (g/L)	1.272 (1.120, 1.489)	**<0.001**	–	–
Creatinine (μmol/L)	0.989 (0.975, 0.998)	0.081	–	–
Myoglobin (ng/mL)	1.000 (0.999, 1.001)	0.786	–	–
CK-MB (ng/mL)	1.018 (0.971, 1.067)	0.436	–	–
hs-cTnI (ng/mL)	1.044 (0.953, 1.143)	0.359	–	–
NT-proBNP (pg/mL)	1.000 (1.000, 1.000)	0.094	–	–
log_2_-NT-proBNP	0.818 (0.647, 1.016)	0.078	0.956 (0.707, 1.289)	0.761
NLR	0.890 (0.802, 0.960)	**0.010**	–	–
PLR	0.998 (0.994, 1.000)	0.180	–	–
SII	1.000 (0.999, 1.000)	0.061	–	–
log_2_-SII	0.559 (0.337, 0.854)	**0.013**	1.070 (0.522, 2.170)	0.850
PNI	1.208 (1.095, 1.368)	**<0.001**	1.163 (1.022, 1.353)	**0.032**

Values with *P* < 0.05 are indicated in bold. “–” in the Multivariable analysis columns indicates that the variable was not included in the predefined multivariable model to maintain conceptual continuity across endpoints or was excluded to avoid multicollinearity among overlapping clinical indices. CI, confidence interval; CK-MB, creatine kinase MB; CRP, C-reactive protein; hs-cTnI, high-sensitivity cardiac troponin I; log_2_-NT-proBNP, log_2_-transformed N-terminal pro-B-type natriuretic peptide; log_2_-SII, log_2_-transformed systemic immune-inflammation index; NLR, neutrophil-to-lymphocyte ratio; NT-proBNP, N-terminal pro-B-type natriuretic peptide; OR, odds ratio; PLR, platelet-to-lymphocyte ratio; PNI, prognostic nutritional index; WBC, white blood cell.

### 3.2 Independent predictors of new embolic events

Baseline characteristics by embolic event status are summarized in Table 1. Patients with new embolic events had larger vegetations (*p* = 0.011), higher WBC count (*p* = 0.026), higher neutrophil count (*p* = 0.043), higher CRP (*p* = 0.045), and higher NLR (*p* = 0.048). Pre-admission antibiotic exposure also differed significantly (*p* = 0.015).

Univariate logistic regression identified vegetation size (OR = 1.198, 95% CI: 1.050–1.409, *p* = 0.014), WBC (OR = 1.177, 95% CI: 1.044–1.353, *p* = 0.012), and neutrophil count (OR = 1.171, 95% CI: 1.039–1.345, *p* = 0.014) as significant predictors (Table 3). To adjust for potential clinical confounders, the predefined baseline panel comprising age, vegetation size, WBC count, log_2_-NT-proBNP, log_2_-SII, and PNI was entered into the final multivariable model. Multivariable analysis showed that adjusting for all covariates, vegetation size (adjusted OR = 1.260, 95% CI: 1.043–1.622, *p* = 0.034) and WBC (adjusted OR = 1.361, 95% CI: 1.097–1.811, *p* = 0.013) remained independent predictors. Age, log_2_-NT-proBNP, log_2_-SII and PNI were not statistically significant.

**TABLE 3:** Univariate and multivariable logistic regression analyses for new embolic events and in−hospital mortality.

Variable	New embolic events (*n* = 13)				In-hospital mortality (*n* = 8)			
	Univariate analysis		Multivariable analysis		Univariate analysis		Multivariable analysis	
	OR (95% CI)	*P*-value	OR (95% CI)	*P*-value	OR (95% CI)	*P*-value	OR (95% CI)	*P*-value
Age (years)	0.996 (0.955, 1.037)	0.837	0.996 (0.927, 1.075)	0.916	1.001 (0.952, 1.053)	0.956	0.988 (0.925, 1.059)	0.724
Weight (kg)	1.018 (0.953, 1.091)	0.591	–	–	0.953 (0.873, 1.028)	0.236	–	–
Vegetation size (mm)	1.198 (1.050, 1.409)	**0.014**	1.260 (1.043, 1.622)	**0.034**	0.926 (0.790, 1.060)	0.295	0.877 (0.701, 1.039)	0.178
WBC count (×10^9^/L)	1.177 (1.044, 1.353)	**0.012**	1.361 (1.097, 1.811)	**0.013**	0.986 (0.836, 1.133)	0.855	0.985 (0.767, 1.248)	0.898
Neutrophil count (×10^9^/L)	1.171 (1.039, 1.345)	**0.014**	–	–	0.981 (0.828, 1.129)	0.802	–	–
Lymphocyte count (×10^9^/L)	0.755 (0.257, 1.979)	0.583	–	–	1.653 (0.519, 5.136)	0.378	–	–
Hemoglobin (g/L)	0.989 (0.963, 1.016)	0.424	–	–	1.023 (0.990, 1.063)	0.192	–	–
Platelet count (×10^9^/L)	0.998 (0.991, 1.004)	0.479	–	–	1.002 (0.995, 1.009)	0.606	–	–
CRP (mg/L)	1.008 (1.000, 1.018)	0.057	–	–	0.997 (0.982, 1.009)	0.687	–	–
Procalcitonin (ng/mL)	0.986 (0.905, 1.017)	0.533	–	–	1.015 (0.985, 1.041)	0.254	–	–
Albumin (g/L)	0.892 (0.784, 1.004)	0.065	–	–	1.025 (0.890, 1.194)	0.734	–	–
Creatinine (μmol/L)	0.991 (0.972, 1.000)	0.232	–	–	0.998 (0.987, 1.003)	0.580	–	–
Myoglobin (ng/mL)	0.999 (0.994, 1.000)	0.550	–	–	1.000 (1.000, 1.001)	0.253	–	–
CK-MB (ng/mL)	1.018 (0.965, 1.066)	0.457	–	–	1.013 (0.948, 1.063)	0.619	–	–
hs-cTnI (ng/mL)	0.987 (0.906, 1.075)	0.767	–	–	1.065 (0.942, 1.204)	0.311	–	–
NT-proBNP (pg/mL)	1.000 (1.000, 1.000)	0.440	–	–	1.000 (1.000, 1.000)	0.955	–	–
log_2_-NT-proBNP	1.113 (0.861, 1.467)	0.424	1.044 (0.643, 1.701)	0.857	1.338 (0.960, 1.987)	0.109	1.651 (1.087, 2.827)	**0.035**
NLR	1.050 (0.993, 1.112)	0.081	–	–	0.964 (0.858, 1.042)	0.434	–	–
PLR	1.001 (0.998, 1.003)	0.505	–	–	1.000 (0.994, 1.002)	0.783	–	–
SII	1.000 (1.000, 1.000)	0.124	–	–	1.000 (1.000, 1.000)	0.923	–	–
log_2_-SII	1.440 (0.900, 2.432)	0.145	0.528 (0.174, 1.390)	0.208	0.831 (0.475, 1.443)	0.506	1.132 (0.482, 2.680)	0.771
PNI	0.925 (0.840, 1.012)	0.099	0.958 (0.786, 1.148)	0.640	1.038 (0.932, 1.168)	0.507	1.127 (0.959, 1.372)	0.177

Values with *P* < 0.05 are indicated in bold. “–” in the Multivariable analysis columns indicates that the variable was not included in the predefined fixed baseline panel model designed to maintain conceptual continuity across clinical endpoints, or was excluded to avoid multicollinearity among overlapping clinical indices. CI, confidence interval; CK-MB, creatine kinase MB; CRP, C-reactive protein; hs-cTnI, high-sensitivity cardiac troponin I; log_2_-NT-proBNP, log_2_-transformed N-terminal pro-B-type natriuretic peptide; log_2_-SII, log_2_-transformed systemic immune-inflammation index; NLR, neutrophil-to-lymphocyte ratio; NT-proBNP, N-terminal pro-B-type natriuretic peptide; OR, odds ratio; PLR, platelet-to-lymphocyte ratio; PNI, prognostic nutritional index; WBC, white blood cell.

### 3.3 Development and validation of the embolic event prediction nomogram

**FIGURE 2:**
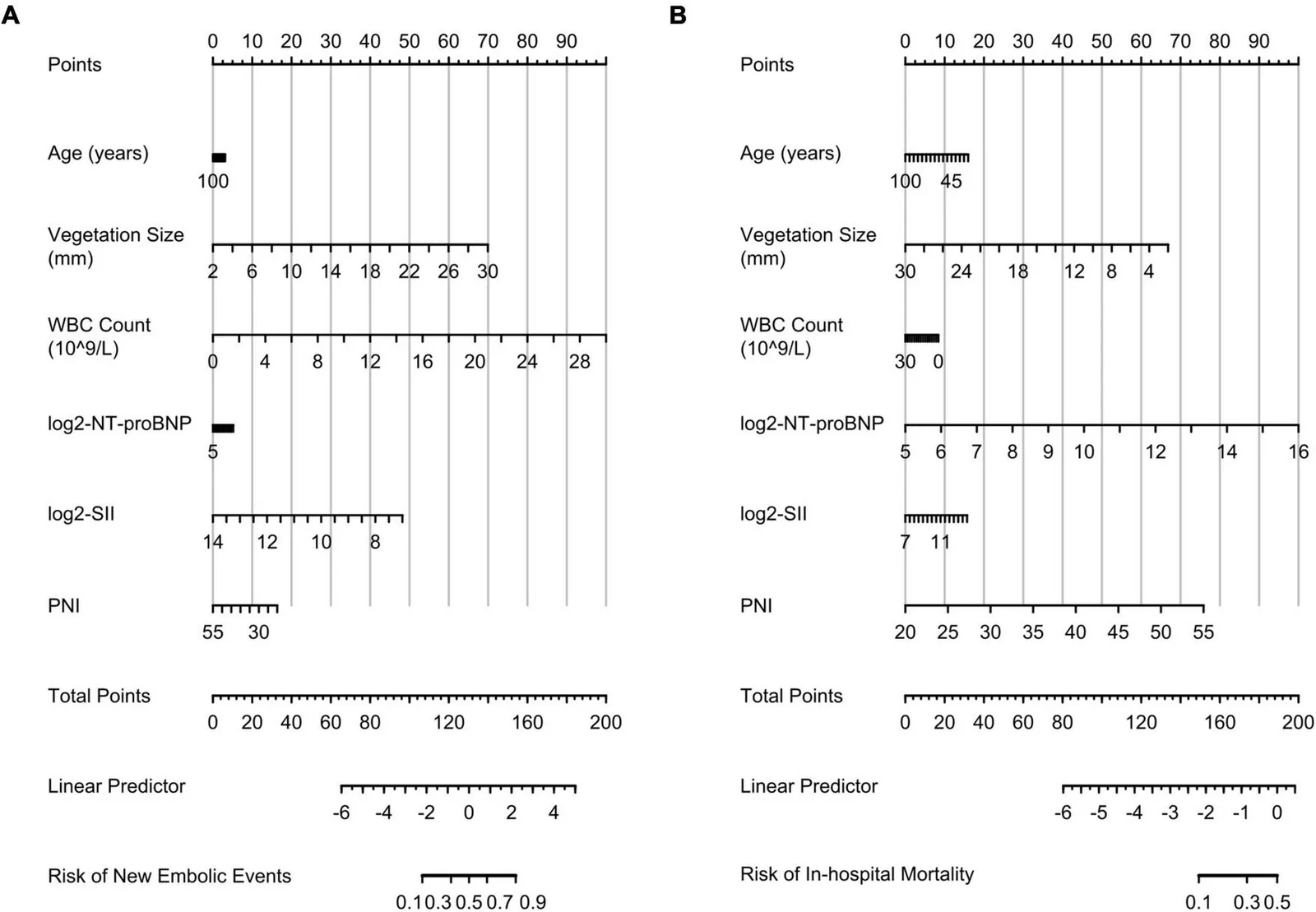
Nomograms for predicting new embolic events and in-hospital mortality in patients with infective endocarditis. **(A)** Nomogram for predicting new embolic events. The model integrates six predictors: age, vegetation size, WBC count, log_2_-NT-proBNP, log_2_-SII, and PNI. Each predictor is assigned a point score on the top scale; the total points are summed and projected onto the bottom scale to estimate the probability of new embolic events. **(B)** Nomogram for predicting in-hospital mortality. The same six predictors (age, vegetation size, WBC count, log_2_-NT-proBNP, log_2_-SII, and PNI) are used to estimate the individual probability of in-hospital mortality. The scoring and probability estimation procedure is identical to that described for panel **(A)**. NT-proBNP, N-terminal pro-B-type natriuretic peptide; PNI, prognostic nutritional index; SII, systemic immune-inflammation index; WBC, white blood cell count.

**FIGURE 3:**
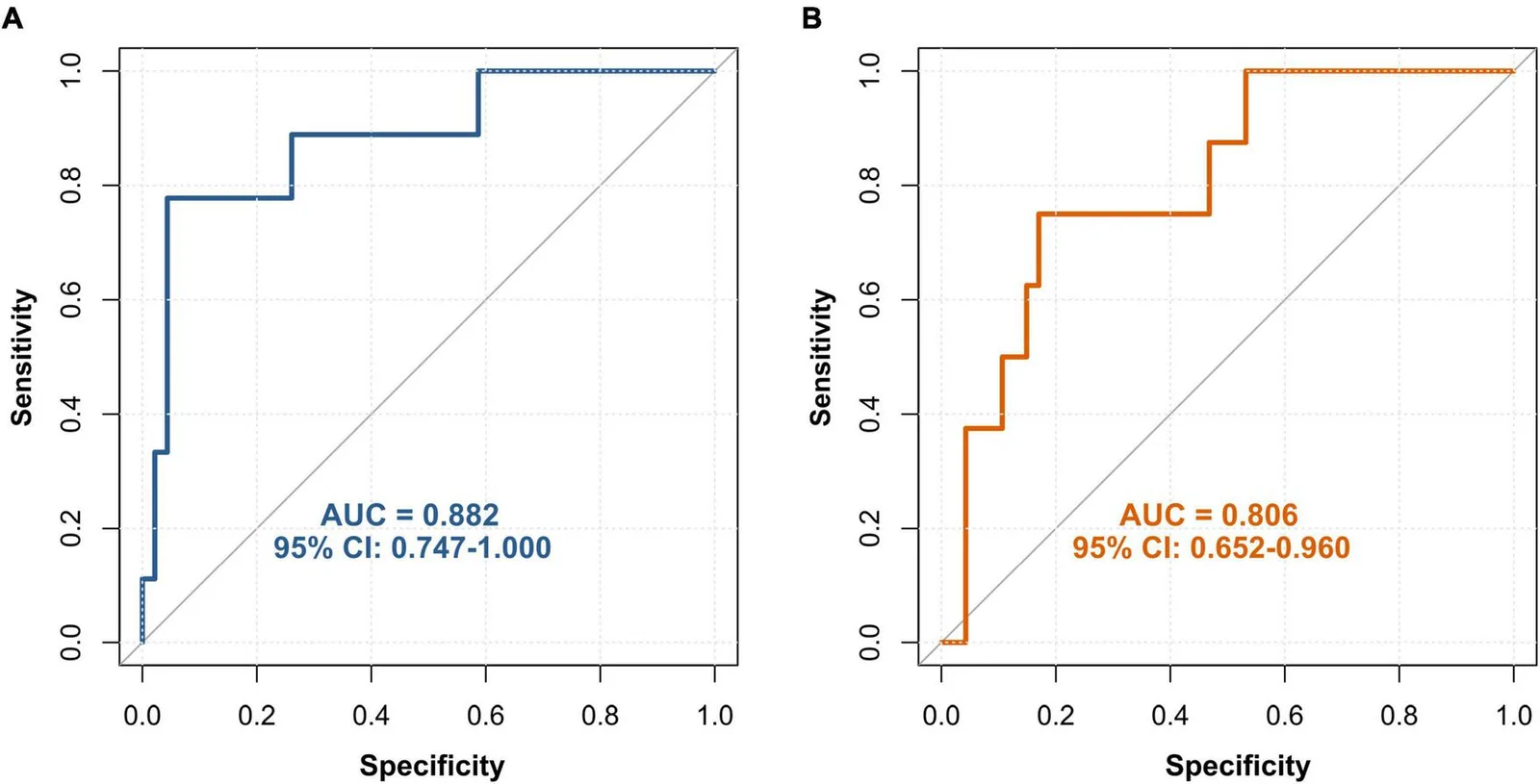
Receiver operating characteristic (ROC) curves for the embolic event and mortality nomograms. **(A)** ROC curve for the 6-predictor embolic event nomogram. The model achieved an area under the curve (AUC) of 0.882. The optimal cut-off value determined by the Youden index was 26.8%, yielding a sensitivity of 77.8% and a specificity of 95.7%. **(B)** ROC curve for the 6-predictor mortality nomogram. The model achieved an AUC of 0.806. The optimal cut-off value was 18.1%, yielding a sensitivity of 75.0% and a specificity of 83.0%.

A multivariable nomogram incorporating all six variables (age, vegetation size, WBC count, log_2_-NT-proBNP, log_2_-SII, and PNI) was constructed to estimate the individual probability of new embolic events (Figure 2A). The model achieved an AUC of 0.882 (Figure 3A). Based on the maximum Youden index, the optimal risk cutoff value was 26.8%, yielding a sensitivity of 77.8% and a specificity of 95.7%.

**FIGURE 4:**
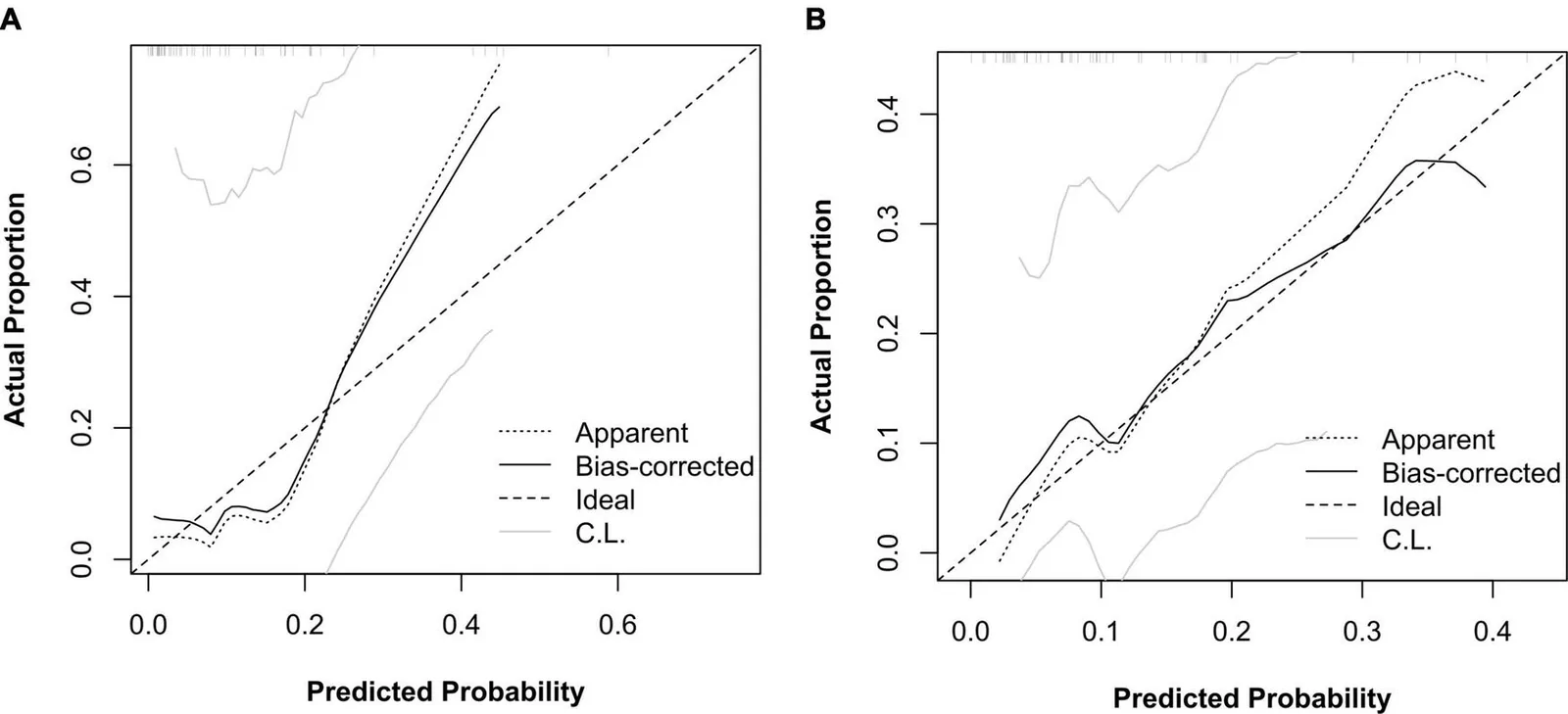
Calibration plots for the embolic event and mortality nomograms. **(A)** Calibration plot for the embolic event nomogram. Predicted probabilities are plotted against observed frequencies, with 1,000 bootstrap resamples for bias correction. The dashed diagonal line represents perfect calibration; the solid curve represents the observed performance. Good agreement between predicted and observed embolic event probabilities was observed across the risk spectrum. **(B)** Calibration plot for the mortality nomogram. Predicted probabilities are plotted against observed in-hospital mortality, with 1,000 bootstrap resamples for bias correction. Satisfactory agreement between predicted and observed mortality probabilities was demonstrated across the risk spectrum.

**FIGURE 5:**
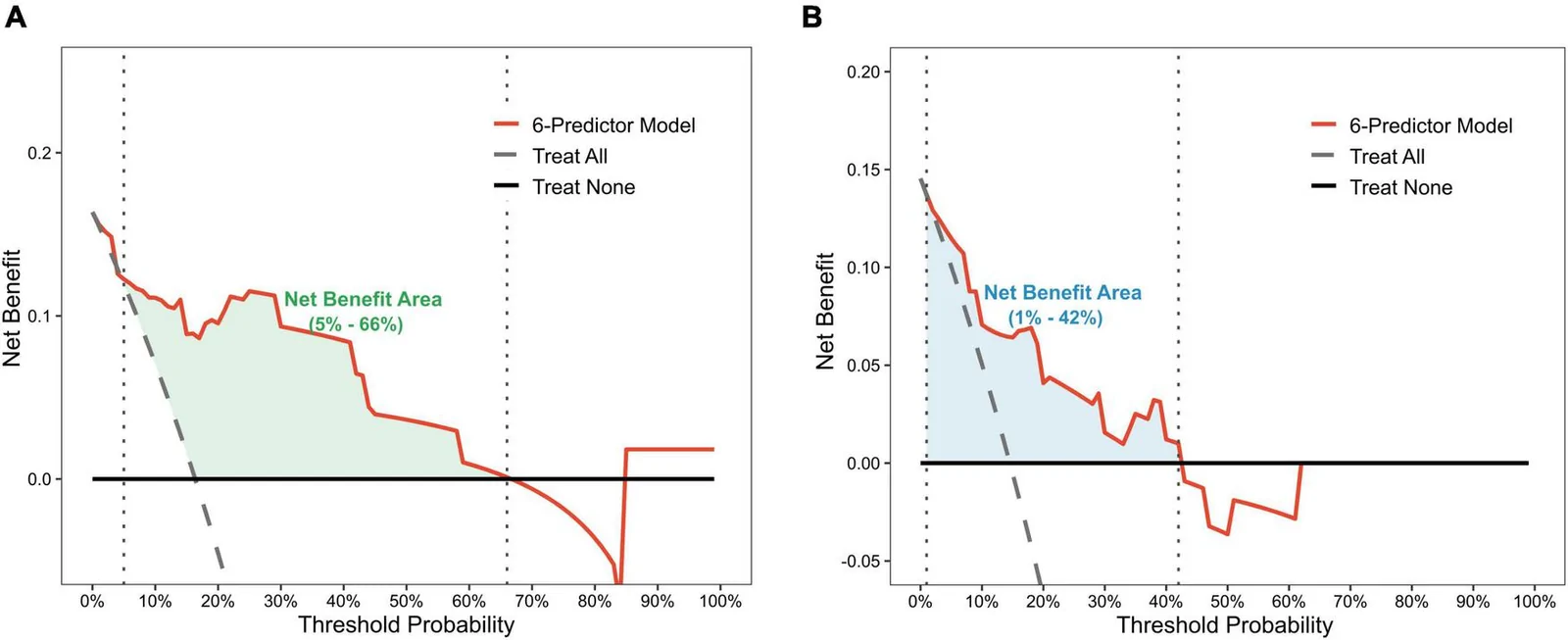
Decision curve analysis (DCA) for the embolic event and mortality nomograms. **(A)** DCA curve for the embolic event nomogram. The *y*-axis represents net clinical benefit, and the *x*-axis represents threshold probability. The 6-predictor nomogram (red curve) demonstrates a positive net benefit compared with both “treat-all” (gray curve) and “treat-none” (black line) strategies across a threshold probability range of 5%–66%. The light green shaded area denotes the net benefit zone where the nomogram yields optimal clinical utility. **(B)** DCA curve for the mortality nomogram. The 6-predictor nomogram (red curve) demonstrates a positive net benefit compared with “treat-all” (gray curve) and “treat-none” (black line) strategies across a threshold probability range of 1%–42%. The light blue shaded area highlights the clinical net benefit zone achieved via the nomogram model.

Calibration with 1,000 bootstrap resamples demonstrated good agreement between predicted and observed embolic event probabilities (Figure 4A). Decision curve analysis for new embolic events demonstrated that the 6-predictor nomogram yielded a positive net clinical benefit compared with both the “treat-all” and “treat-none” strategies across a threshold probability range of 5%–66% (Figure 5A), suggesting potential clinical utility for risk-stratified intervention.

### 3.4 Independent risk factors for In-Hospital mortality

Baseline characteristics by survival status are presented in Table 1. Non-survivors were more likely to have NYHA class III–IV symptoms (*p* = 0.040). No significant differences were observed in WBC count, neutrophil count, or CRP between survivors and non-survivors (all *p* > 0.050).

Univariate and multivariable logistic regression analyses were subsequently performed to determine independent predictors (Table 3). Following multivariable adjustment of the six designated candidate variables, log_2_-NT-proBNP emerged as the sole statistically significant independent predictor of in-hospital mortality (adjusted OR = 1.651, 95% CI: 1.087–2.827, *p* = 0.035). The remaining clinical and laboratory covariates, including age, vegetation size, WBC, log_2_-SII, and PNI, were retained in the final model but did not reach statistical significance (all *p* > 0.050; details in Table 3).

### 3.5 Development and validation of the mortality prediction nomogram

A nomogram incorporating age, vegetation size, WBC count, log_2_-NT-proBNP, log_2_-SII and PNI was developed to estimate the individualized probability of in-hospital mortality (Figure 2B). The model achieved an AUC of 0.806 (Figure 3B). Based on the maximum Youden index, the optimal cut-off value was 18.1%, with a sensitivity of 75.0% and a specificity of 83.0%.

Calibration with 1,000 bootstrap resamples demonstrated satisfactory agreement between the predicted and observed mortality probabilities (Figure 4B). Decision curve analysis indicated that the nomogram provided a positive net clinical benefit compared with the “treat-all” and “treat-none” strategies, across a threshold probability range of 1%–42% (Figure 5B).

## 4 Discussion

In this retrospective cohort study of adult patients with IE, we developed and internally evaluated two exploratory, 6-variable nomogram-based prediction frameworks (Figure 2). Given our modest sample size, these models are presented not as definitive diagnostic algorithms, but as hypothesis-generating aids for preliminary risk stratification. Our results tentatively suggest distinct pathophysiological pathways within this cohort: short-term mortality appears to be linked to severe cardiac hemodynamic stress, as estimated by log_2_-NT-proBNP levels, which aligns with previous evidence regarding neurohormonal activation in acute valvular disruption (Kahveci et al., 2007; Bertolino et al., 2022). Conversely, new embolic events seem to involve a combined mechanical-immunological interplay represented by vegetation size and systemic leukocyte burden (WBC count), consistent with established paradigms on clot stability and inflammation-induced friability (Thuny et al., 2005; Bertolino et al., 2025). Both frameworks demonstrated acceptable discrimination, satisfactory calibration, and potential clinical utility within designated risk threshold ranges (Figures 3–5), indicating that routinely accessible bedside variables warrant further large-scale investigation for individualized risk assessment.

The observed association between elevated baseline NT-proBNP and in-hospital mortality is consistent with the recognized pathophysiology of acute valvular disruption. Acute endocarditis-induced valvular devastation frequently precipitates severe regurgitation, volume overload, and myocardial wall stress, which collectively trigger the synthesis and release of natriuretic peptides (Bertolino et al., 2022). Notably, within our final adjusted multivariable model, only log_2_-NT-proBNP retained statistical significance for the mortality endpoint, whereas the nutritional and immunothrombotic indices (PNI and SII) did not. This preliminary observation suggests that once structural cardiac damage has occurred in the acute phase, short-term survival within this specific cohort might be more closely tied to hemodynamic compensation and remaining myocardial reserve than to the magnitude of systemic inflammation alone, a hypothesis that aligns with established multicenter cohorts demonstrating the overriding prognostic impact of acute cardiac dysfunction over inflammatory state (Bohbot et al., 2022; Lozano Ibañez et al., 2024). This conceptual framework further aligns with broader clinical literature highlighting heart failure as a primary driver of adverse outcomes in IE, where early cardiac biomarker evaluations may offer essential indicators of impending hemodynamic instability (Nishimura et al., 2017; Habib et al., 2019).

Regarding thromboembolic complications, our results provide supportive evidence for a potential “mechanical-immunological” dual-risk hypothesis (Thuny et al., 2005; Liesenborghs et al., 2020). This conceptual framework posits that embolic events in IE arise from either mechanical dislodgement of the vegetation under intracardiac shear stress or inflammatory destabilization mediated by host immune activation (Engelmann and Massberg, 2013; Serban et al., 2025). Echocardiographic vegetation size has historically been utilized as a primary marker of embolic risk, given that larger or highly mobile vegetations possess a greater propensity for mechanical fragmentation under the shear stress of intracardiac blood flow (Mohananey et al., 2018). Interestingly, our multivariable analysis suggests that this structural risk might be synergistically compounded when accompanied by an elevated WBC count. Clinically, in the acute phase of IE, an elevated total WBC count is predominantly driven by the massive mobilization of circulating neutrophils (Manz and Boettcher, 2014). From a cellular perspective, these neutrophils actively infiltrate the vegetation matrix (Meyers et al., 2023); emerging molecular evidence indicates that through the active release of proteolytic enzymes and NETs, these cells can systematically degrade the local fibrin scaffold, thereby destabilizing the core vegetation architecture and facilitating thromboembolic shedding (Fuchs et al., 2010; Döring et al., 2017). The 6-variable nomogram–which integrates vegetation size and WBC count along with age, log_2_-NT-proBNP, log_2_-SII, and PNI–achieved an AUC of 0.882, providing preliminary support for the hypothesis that combining macroscopic structural imaging with basic systemic cellular immune profiles may refine risk stratification for embolic events (Hubert et al., 2013).

Furthermore, although age, log_2_-SII, and PNI did not reach statistical significance in the final adjusted models, they were retained to preserve the framework’s conceptual completeness. Variable selection and the exclusion of post-diagnosis treatments (such as early surgery) should be guided by clinical relevance and the prevention of selection bias, rather than statistical significance alone (Collins et al., 2015). IE is inherently a systemic illness; comprehensive indices like PNI and SII encapsulate the patient’s baseline immunothrombotic and nutritional reservoir, reflecting complementary dimensions of systemic host pathophysiology (Hu et al., 2022; Abulimiti et al., 2025). Notably, our exploratory descriptive analysis revealed that a younger age and a higher PNI were closely tied to a greater likelihood of undergoing surgical intervention. Because real-world surgical decisions are heavily driven by clinicians’ subjective perceptions of operative tolerance (Kang et al., 2012), incorporating surgery would introduce a profound treatment-indication bias. Consequently, surgical intervention was intentionally omitted from the models to preserve their prognostic validity.

From a clinical translation perspective, DCA substantiated the potential utility of these exploratory nomograms within specific clinical threshold ranges. For the embolic prediction framework, a continuous positive net benefit was observed across a threshold probability continuum from 5% to 66%. This interval provides a valuable reference range for clinicians balancing the risk of embolic stroke against the bleeding hazards of antithrombotic therapies in patients with IE–a scenario where therapeutic anticoagulation or antiplatelet management remains a formidable clinical dilemma (Caldonazo et al., 2024). Similarly, the mortality nomogram demonstrated a positive net benefit across a practical threshold range of 1%–42%. These preliminary ranges suggest that our integrated model scoring could help shield low-risk patients from unnecessary, invasive interventions while capturing highly vulnerable individuals who require intensive monitoring, aligning with the objectives of established IE risk stratification tools (Park et al., 2016). Nevertheless, these thresholds require external validation in independent prospective cohorts before clinical implementation, as mandated by contemporary guidelines (Collins et al., 2015).

When interpreting these findings, several inherent limitations must be emphasized. First, the retrospective, single-center design is susceptible to selection bias and restricts the broad generalizability of our findings. Additionally, our cohort exhibited a high culture-negative rate (48.4%) driven by pre-admission antibiotic exposure (74.2%), alongside the absence of advanced molecular testing (e.g., mNGS or intracellular PCR), and a treatment-indication bias where younger, nutritionally robust patients (higher PNI) were preferentially selected for surgery. Nevertheless, these real-world constraints reflect local tertiary practice and underscore the utility of culture-independent, immuno-nutritional risk stratification, while multivariable modeling identified independent associations of age and PNI with surgical management. Second, and most importantly, the sample size was comparatively modest (*N* = 62), and the number of primary outcome events–particularly in-hospital mortality (*n* = 8)–was highly limited. This resulted in a very low events-per-variable (EPV) ratio for our 6-variable multivariable analyses. Methodological literature demonstrates that such low EPV ratios substantially amplify the risk of model overfitting and restrict the statistical power required to detect smaller, more subtle independent prognostic effects (Peduzzi et al., 1996). This statistical constraint likely explains why several comprehensive indices (such as log_2_-SII, PNI, and age) did not achieve statistical significance within the multivariable models, despite their established biological plausibility. Consequently, our nomograms must be interpreted strictly as exploratory, hypothesis-generating frameworks rather than definitive clinical decision-making tools. The nomograms are intended to complement, rather than replace, established guideline-based risk assessment incorporating clinical and echocardiographic predictors. NT-proBNP may provide additional information on cardiac stress and potentially support closer monitoring or multidisciplinary assessment, although whether its incorporation directly changes management remains to be determined. Finally, rigorous external validation in larger, multicenter, prospective cohorts remains an essential prerequisite before routine clinical implementation can be considered.

In conclusion, within the scope of this exploratory study, elevated log_2_-NT-proBNP was independently associated with in-hospital mortality, while echocardiographic vegetation size and WBC count emerged as independent predictors of new embolic events. Integrating these readily accessible variables into our 6-variable nomograms provides a preliminary, proof-of-concept framework for individualized bedside risk stratification. However, given the modest sample size and the exploratory nature of this work, subsequent large-scale, multicenter external validation remains an essential prerequisite before any potential clinical translation.

## Data Availability

The raw data supporting the conclusions of this article will be made available by the authors, without undue reservation.

## Supplementary material

Supplementary Material 1 TRIPOD checklist for prediction model development and validation.
